# Narrative enhancement and cognitive therapy (NECT) to improve social functioning in people with serious mental illness: study protocol for a stepped-wedge cluster randomized controlled trial

**DOI:** 10.1186/s13063-021-05067-1

**Published:** 2021-02-08

**Authors:** J. Dubreucq, M. Faraldo, M. Abbes, B. Ycart, H. Richard-Lepouriel, S. Favre, F. Jermann, J. Attal, M. Bakri, T. Cohen, C. Cervello, I. Chereau, C. Cognard, M. De Clercq, A. Douasbin, J. Y. Giordana, E. Giraud-Baro, N. Guillard-Bouhet, E. Legros-Lafarge, M. Polosan, A. Pouchon, M. Rolland, N. Rainteau, C. Roussel, C. Wangermez, P. T. Yanos, P. H. Lysaker, N. Franck

**Affiliations:** 1 Centre de Neurosciences Cognitive, UMR 5229, CNRS & Université Lyon 1, Lyon, France; 2 Centre Référent de Réhabilitation Psychosociale et de Remédiation Cognitive (C3R), Centre Hospitalier Alpes Isère, 1 place du Conseil National de la Résistance, 38400 Saint Martin d’Hères, Grenoble, France; 3 Fondation FondaMental, Créteil, France; 4 Réseau Handicap Psychique, Grenoble, France; 5 Laboratoire Jean Kuntzmann, CNRS UMR 5224, Université Grenoble-Alpes, Grenoble, France; 6 Department of Psychiatry, Mood disorders Unit, Geneva University Hospital, 20bis rue de Lausanne, CH-1201 Geneva, Switzerland; 7 Service Universitaire de Psychiatrie Adulte, Hôpital la Colombière, CHRU Montpellier, Université Montpellier 1, Inserm, 1061 Montpellier, France; 8 Centre de Réhabilitation Psychosociale et de Remédiation Cognitive (C2R), CH Drôme Vivarais, Montéléger, France; 9 Centre Ressource de Réhabilitation Psychosociale et de Remédiation Cognitive, Centre Hospitalier Le Vinatier, Bron, France; 10 Centre Référent Lyonnais de Réhabilitation Psychosociale et de Remédiation Cognitive (CL3R), Centre Hospitalier Le Vinatier, Bron, France; 11 CMP B, CHU, EA 7280 Faculté de Médecine, Université d’Auvergne, BP 69 63003 Clermont-Ferrand Cedex 1, France; 12 Unité Ariane de rehabilitation psychosociale, EPSM, Caen, France; 13 Centre Départemental de Réhabilitation Psychosociale des Glières, 219 chemin des bois des Fornets, 74800 La Roche sur Foron, France; 14 Centre Hospitalier Sainte Marie de Nice, 87 Avenue Joseph Raybaud, 06100 Nice, France; 15 Clinique du Dauphiné- Groupe Sinoué, 252 Route de Saint-Nizier, 38180 Seyssins, France; 16 CREATIV & URC Pierre Deniker, CH Laborit, Poitiers, France; 17 Centre Référent de Réhabilitation Psychosociale de Limoges C2RL, CH Esquirol, Limoges, France; 18 Centre Expert Troubles Bipolaires, Service Universitaire de Psychiatrie, CHU de Grenoble et des Alpes, CS10217, F-38043 Grenoble, France; 19 John Jay College of Criminal Justice, City University of New York, New York, USA; 20 Roudebush VA Medical Center, Indiana University School of Medicine, Indianapolis, USA

## Abstract

**Background:**

Self-stigma is highly prevalent in serious mental illness (SMI) and is associated with poorer clinical and functional outcomes. Narrative enhancement and cognitive therapy (NECT) is a group-based intervention combining psychoeducation, cognitive restructuring and story-telling exercises to reduce self-stigma and its impact on recovery-related outcomes. Despite evidence of its effectiveness on self-stigma in schizophrenia-related disorders, it is unclear whether NECT can impact social functioning.

**Methods:**

This is a 12-centre stepped-wedge cluster randomized controlled trial of NECT effectiveness on social functioning in SMI, compared to treatment as usual. One hundred and twenty participants diagnosed with schizophrenia, bipolar disorder or borderline personality disorder will be recruited across the 12 sites. The 12 centres participating to the study will be randomized into two groups: one group (group 1) receiving the intervention at the beginning of the study (T0) and one group (group 2) being a control group for the first 6 months and receiving the intervention after (T1). Outcomes will be compared in both groups at T0 and T1, and 6-month and 12-month outcomes for groups 1 and 2 will be measured without a control group at T2 (to evaluate the stability of the effects over time). Evaluations will be conducted by assessors blind to treatment allocation. The primary outcome is personal and social performance compared across randomization groups. Secondary outcomes include self-stigma, self-esteem, wellbeing, quality of life, illness severity, depressive symptoms and personal recovery.

**Discussion:**

NECT is a promising intervention for reducing self-stigma and improving recovery-related outcomes in SMI. If shown to be effective in this trial, it is likely that NECT will be implemented in psychiatric rehabilitation services with subsequent implications for routine clinical practice.

**Trial registration:**

ClinicalTrials.gov NCT03972735. Trial registration date 31 May 2019.

## Introduction

Mental illness stigma refers to the negative beliefs, emotional reactions and attitudes towards people with serious mental illness (SMI) endorsed by the general population (also referred to as public stigma [1]). People with SMI are generally acutely aware of public stigma and expect to be rejected by others (69.4% of the 1229 participants with schizophrenia (SZ) and 71.6% of the 1182 participants with mood disorders in the GAMIAN-Europe study had high perceived stigma [2, 3]). Self-stigma occurs when someone moves beyond awareness of stigma to accepting the negative stereotypes about SMI as true to describe him/herself [1]. Self-stigma refers to the process whereby a person’s previously held social identity (defined by social roles such as son, brother, sister, friend, employee or potential partner) is progressively replaced by a devalued and stigmatized view of oneself. Self-stigma is highly frequent in Europe (41.7% in SZ and 21.7% in mood disorders [2, 3]) and the USA (36.1% out of 144 people with SMI [4]). The ‘illness identity model’ [5] proposed that self-stigma can have pervasive effects on outcomes related to recovery from SMI, including self-esteem, hopefulness, social interaction, employment and symptom severity. The evidence consistently supports many of the model’s predictions, including that self-stigma is negatively associated with self-esteem, motivation to achieve personal life goals, shared-decision making, adherence into treatment, well-being, quality of life, personal recovery and social function [6–14]. People with elevated self-stigma report more dysfunctional attitudes, social withdrawal, depressive symptoms and increased suicidal ideation [5, 15–21]. High insight into illness directly predicts and compounds the effects of self-stigma on depression [15, 17, 22–24]. Impairments in cognitive functioning, metacognition and social cognition predict increased self-stigma [12, 24–29].

Several psychosocial interventions have been designed to reduce self-stigma and its impact on patient’s outcomes, with preliminary results on self-stigma, insight and self-efficacy [30]. Narrative enhancement and cognitive therapy (NECT) is a structured, 20 session group-based approach which combines psychoeducation to counteract SMI stereotypes, cognitive restructuring to challenge dysfunctional attitudes about the self and story-telling exercises to enhance meaning-making and the person’s ability to move beyond their illness-identity to a more integrated sense of self [31]. NECT is manualized and can be implemented with fidelity in routine treatment settings [31]. Four controlled studies, including three randomized controlled trials (RCT) in three different countries (USA, Israel, Sweden) have been published to date in psychotic-related disorders. NECT was effective in improving self-stigma, hope and self-esteem [32–34] with persisting effects after 6 months’ follow-up [33, 34]. The results on subjective quality of life were mixed, one study found improvements [32] but another found no effectiveness [33]. NECT was effective in reducing social withdrawal and avoidant coping strategies in participants with schizophrenia (SZ) in comparison with an active control condition [34]. However, NECT was not effective on social functioning, measured with the Quality of Life Scale [35], a global measure of psychosocial function [34]. In almost all studies of NECT, the samples were mixed, with some participants showing low levels of self-stigma (Internalized Stigma of Mental Illness (ISMI) total score < 2.5) and others moderate to high self-stigma (ISMI > 2.5). Changes in coping strategies were higher in participants from centres providing the intervention within the context of psychiatric rehabilitation [34].

In summary, NECT has demonstrated effectiveness on self-stigma and subjective aspects of recovery (hope, self-esteem and quality of life) in participants with psychotic-related disorders and SZ. Its effectiveness on social function and in other stigmatized SMI conditions (bipolar disorder (BD), borderline personality disorder (BPD)) are, however, still unclear. Similarly, NECT’s effectiveness in improving depression, treatment adherence, wellbeing and personal recovery is still unknown.

The objectives of the present study are to investigate (i) NECT effectiveness on social functioning in SMI participants, (ii) NECT effectiveness on self-stigma, psychiatric symptoms, depression, well-being, subjective quality of life and personal recovery, (iii) whether insight into illness and cognitive functioning at baseline predict improvements at follow-up and (iv) whether the effects on primary and secondary outcomes persist after 6 and 12 months’ follow-up.

The primary hypothesis to be tested is whether NECT significantly improves social functioning in comparison with the control group. The secondary hypotheses are to determine if, when compared to the control condition, NECT results in significant improvements in self-stigma, psychiatric symptoms, depression, wellbeing, self-esteem, subjective quality of life and personal recovery.

## Methods and design

### Design

**Fig. 1:**
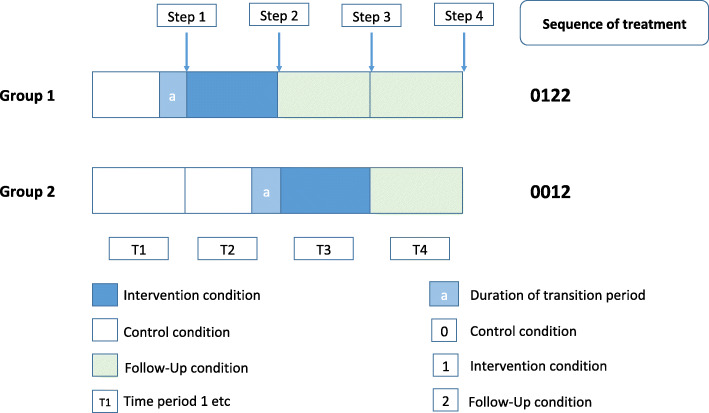
Diagram of the stepped-wedge trial design

This stepped wedge cluster randomized controlled study is multi-centric, prospective, interventional and exploratory. For ethical purposes, we have chosen a stepped wedge cluster randomized trial design, with a waiting list type control condition. Previous research from the French national REHABase network showed that (i) most patients were dissatisfied with their interpersonal relationships at the time of admission and asked for interventions improving social function [36] and (ii) elevated self-stigma was frequent (31.2% of the 738 participants [37]) and was negatively associated with recovery-related outcomes. Self-stigma has been negatively associated with patient’s longitudinal outcomes and to lower benefits from psychiatric rehabilitation [38]. Providing NECT to all participants aims at not excluding participants from interventions improving social function (e.g. social skills training or social-cognitive remediation) during the follow-up period without proposing them a credible therapeutic alternative (i.e. self-stigma reduction). This also aims at reducing potential deception bias and dropouts in participants allocated to the control group. Each centre will represent a cluster. In stepped-wedge RCTs, all clusters are evaluated before and after providing the intervention and each cluster switches from control to become exposed, but not at the same point in time [39, 40]. The 12 centres participating to the study will be randomized into two groups: one group (group 1) receiving the intervention at the beginning of the study (T0) and one group (group 2) being a control group for the first 6 months and receiving the intervention after (T1). The stepped-wedge design of this 2-group trial can be seen on Fig. 1.

**Fig. 2:**
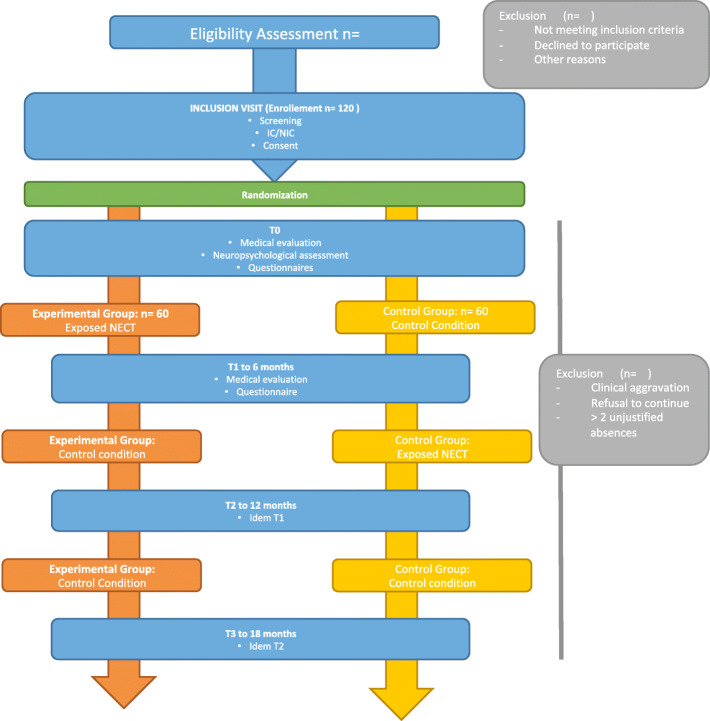
Schematic overview of the study protocol

The total duration of the study will be 18 months to allow for recruitment of the target number of participants, delivery of the intervention, follow-up assessments and data analysis using intention-to-treat methods. A schematic overview of the study design is presented in Fig. 2.

### Participants

#### Inclusion and exclusion criteria

Eligible participants will be adults (age > 18) diagnosed with schizophrenia, schizoaffective disorder, schizotypal disorder, schizophreniform disorder, bipolar I or II disorder or borderline personality disorder (DSM-5 criteria [41]). For the purpose of the study only stabilized outpatients will be recruited (defined as 3 months of clinical stability and no changes in pharmacological treatment in the past 3 months). Participants unable to give informed consent or with a comorbid intellectual disability will be excluded. Participation in other programs having an impact on social function or self-stigma (social cognitive remediation, social skills training) will be prohibited during the follow-up period.

#### Recruitment and randomization

**Fig. 3:**
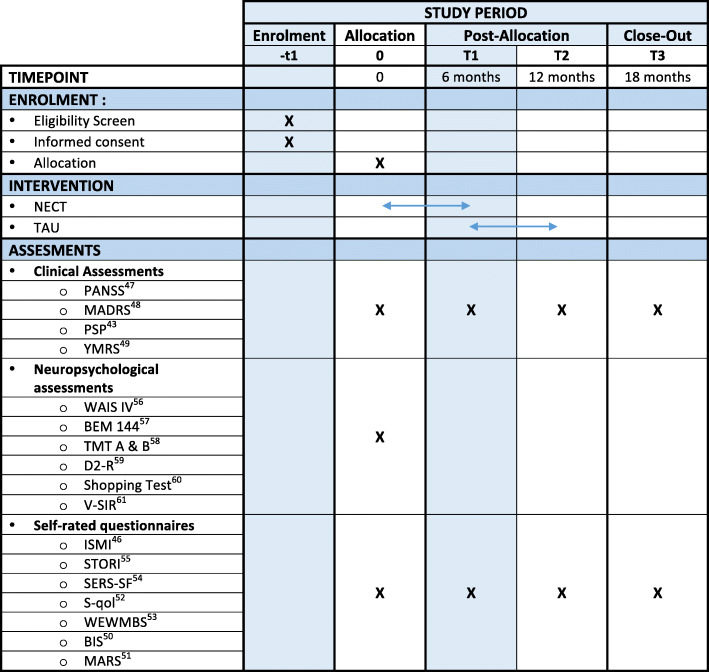
Schedule of enrolment, interventions and assessments (SPIRIT diagram). BIS, Birchwood Insight Scale; ISMI, Internalized Stigma of Mental Illness; MARS, Medication Adherence Rating Scale; NECT, narrative enhancement and cognitive therapy; PSP, Personal and Social Performance Scale; S-QoL, Subjective-Quality of Life; TAU, treatment as usual; TMT, Trail Making Test; V-SIR, Versailles-Situational Intention Reading; WAIS IV, Wechsler Adult Intelligence Scale-4th edition; WEWMBS, Warwick-Edinburgh Mental Wellbeing Scale; YMRS, Young Mania Rating Scale

Patients diagnosed with schizophrenia, bipolar disorder or borderline personality disorder (DSM-5 criteria [41]) will be recruited through six Rehabase psychiatric rehabilitation network centres [36], three FondaMental Academic Centres of Expertise (FACE) for SZ or BD [42, 43], one unit specialized in mood disorders at the Geneva university hospital, one psychiatric day-care clinic specialized in mood and personality disorders in Grenoble and one psychiatric rehabilitation centre located in the psychiatric hospital in Caen. The selected sites are already actively involved in treating patients with SZ, BD or BPD and have previous research experience in the field of psychiatric rehabilitation. Regular group meetings will be organized to monitor quality control and ensure good inter-rater reliability. A list of eligible participants will be generated under the supervision of the coordinating centre clinical research officer. Each eligible patient will be approached by a trained member of the research team and invited to participate in the study, thereby eliminating selection biases. Eligible patients will be given a leaflet describing the program and the research. Informed consent will be taken during a medical interview with one of the investigators. The clinical research officer of the coordinating centre (Grenoble) will be in charge of the randomization process for participants from all centres. Clusters will be randomized to receive the intervention at either T0 or T1. Stratified randomization will be used to ensure that cluster types are well balanced across groups. Outcomes will be compared in all groups at T0 and T1 (duration of intervention= 6 months). Six-month and 12-month outcomes for groups 1 and 2 will be measured at T2 (18 months after T0) to evaluate the stability of the effects over time. A 7-point improvement in social functioning measured using Personal and Social Performance Scale [44] (PSP) is generally considered as clinically significant. We plan to include 5 patients per centre and per group, meaning that there will be 10 participants included per centre for a total of 120 patients included (60 in group 1, 60 in group 2). This sample size was calculated using R package ‘pwr’ [45] based on Cohen [46] (*d* = 0.50, alpha level of 0.05) without allowing for intra-cluster correlations and the cluster and time effects found in stepped-wedge designs [47, 48]. This could be a considerable limitation, as it might have underpowered the trial [47]. Most of the outcome measures are self-rated questionnaires; the other evaluations will be conducted by an evaluator who has not taken part in the intervention. The schedule of enrolment, interventions and assessments is shown in Fig. 3 (SPIRIT diagram).

### NECT

#### Translation procedure

A consistent translation and cross-cultural adaptation procedure was realized by two independent research teams (JD, MF and MA in Grenoble; HRL, SF and FJ in Geneva). All materials were translated from English into French, the first language of all translators. Each team reviewed the material translated by the other team so all translations were crosschecked. Regular group meetings between the two teams were conducted to discuss any problematic translations and resolve any disputed items, in line with established translation methods.

#### Training and fidelity monitoring

All the clinicians involved will attend a 1-day NECT training session, delivered by the creators of the program (Yanos PT, Roe D). Training will include discussion and role-playing exercises, with corrective feedback from the trainers. Regular group supervisions will be organized to ensure treatment fidelity. NECT fidelity will be monitored using the NECT Fidelity Scale, developed by Yanos et al. [34].

#### The experimental intervention

**Fig. 4:**
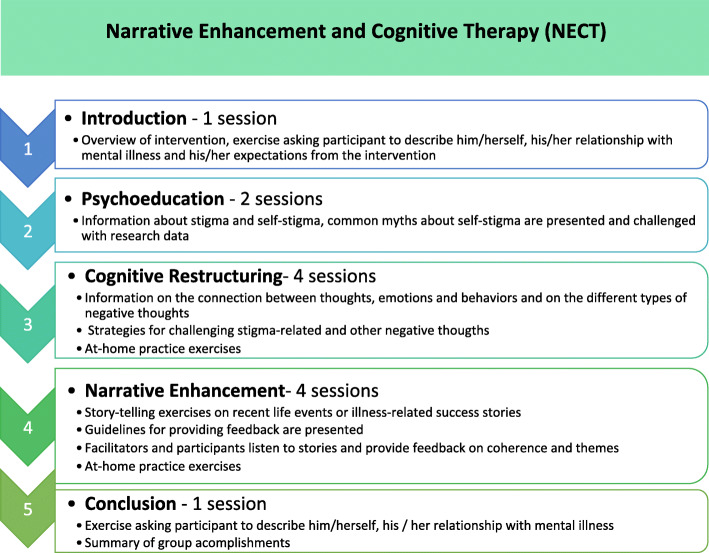
Structure of NECT topics

The French adaptation of NECT is a manualized, structured, 12-session, group-based intervention conducted by two facilitators. The number of sessions was reduced in the French adaptation and their duration extended to match the typical duration of psychosocial interventions in French-speaking countries (up to 15 sessions). Figure 4 provides an overview of the NECT topics. The 2-h sessions are divided into three main parts: (1) psychoeducation about stigma, self-stigma and myths about mental disorders; (2) cognitive restructuring targeting negative thoughts about the self and dysfunctional attitudes and (3) story-telling exercises in which participants tell stories about themselves, receive feedback from other group members and facilitators and are invited to apply the cognitive restructuring skills learnt in the previous sessions to their personal life narratives. At-home practice exercises are carried out between the sessions.

### The control condition

The control condition refers to the group receiving current standard care during the first 6 months of the study before attending NECT after T1. There is no definition of standard care. The association of pharmacological treatment with psychiatric rehabilitation is however recommended in all the international guidelines on SMI [49–51]. All participants will attend day-care recovery-oriented services offering several forms of psychiatric rehabilitation (group CBT on positive symptoms, general psychoeducation, cognitive remediation, vocational rehabilitation, supported employment). Information about the interventions received during the follow-up period (with their duration and the number of sessions) will be systematically collected by the clinical research officer and taken into account when analysing the results.

### Assessments

At baseline and at follow-up, socio-demographic and illness-related information (education level, employment and marital status, psychiatric diagnosis, age of onset, illness duration, current pharmacological treatment) will be collected. All outcome measures listed below are part of routine clinical evaluations in the national REHABase and FACE networks for SZ and BD [36, 42, 43] approved by the relevant ethical committees.

#### Primary outcome

Social function will be assessed with the Personal and Social Performance Scale [44] (PSP), a clinician-reported measure of social dysfunction using six-point severity scale in four life domains (socially useful activities, personal and social relationships, self-care and disturbing or aggressive behaviours). PSP provides an overall rating score ranging from 1 to 100, higher scores representing better personal and social functioning. PSP showed acceptable internal consistency [52], excellent inter-rater reliability [53] and a satisfactory ability to detect changes [44]. A seven-point improvement during clinical trials is considered to be clinically significant [44].

#### Secondary outcomes

Self-stigma will be assessed using the Internalized Stigma of Mental Illness scale (ISMI [2, 54]), a 29-item self-report measure designed to assess a person’s personal experience of stigma related to mental disorders and is rated on a four-point Likert scale. Items are summed to provide a mean total score and five subscale scores (alienation or feeling of being a devalued member of the society; stereotype endorsement or agreement with the negative attitudes about SMI; discrimination experience; social withdrawal as a coping strategy and stigma resistance). Stigma resistance is generally excluded because of poor correlations with other subscales [32, 34]. Higher scores reflect higher levels of self-stigma. Scores above 2.5 indicate moderate to high levels of self-stigma [2, 3]. Illness severity will be assessed in patients with SZ using the Positive and Negative Syndrome [55] (PANSS) scales. Current depressive symptoms were evaluated using the Montgomery-Asberg Depression Rating Scale [56] (MADRS) and current manic symptoms with the Young Mania Rating Scale [57] (YMRS). Insight and treatment adherence will be assessed with self-reported measures (Birchwood Insight Scale (BIS) [58]; Medication Adherence Rating Scale (MARS) [59]). Quality of life will be evaluated with the self-reported Subjective Quality of Life scale [60] (S-QoL) and wellbeing using the Warwick-Edinburgh Mental Well-being Scale [61] (WEMBS). Self-esteem will be measured with the Self-Esteem Rating Scale [62] (SERS) and personal recovery using the self-reported Stage of Recovery Instrument [63] (STORI). Baseline neuropsychological cognitive assessments include Wechsler Adult Intelligence Scale-4th edition [64] (WAIS-IV) subscales assessing working memory, verbal abstraction and hypothetico-deductive reasoning, BEM-144 story subtest for auditive-verbal memory [65], Trail Making Test A and B (TMT-A or B) [66] respectively for speed of processing and reactive mental flexibility, D2-R for selective attention, concentration and speed of processing [67], and shopping test for planning abilities [68]. Theory of mind will be assessed at baseline using the Versailles-Situational Intention Reading Test [69] (V-SIR).

### Statistical analysis

Intention-to-treat analyses will be conducted in the first instance. The analyses will be conducted using parametric tests for normal distributed data (Student’s *t* and covariance analysis with repeat measures ANCOVA) and with non-parametric tests (Mann-Whitney *U* and Kruskal-Wallis test) in case of significant deviations from the normal distribution. The primary analysis was done by intention to treat.

We will analyse all outcomes using multilevel regression models with time point and intervention status as fixed effects and clusters as a random effect. Timepoint will be included as a categorical variable [39]. The statistical analysis will be done using the R software, version 3.2.3 [70]. The psych package version 1.5.8 will be used [71]. Effect size (Cohen’s *d*) will be calculated using the effsize package [71]. Size effects inferior to 0.20 will be considered as negligible, from 0.20 to 0.40 as small, from 0.40 to 0.60 as moderate and superior to 0.60 as strong [71]. The leaps package version 2.9 will be used for variable selection [72]. The level of confidence intervals will be set at 0.95 and the significance level of tests at 0.05.

## Trial status

The present trial has not yet started. The recruitment will begin on September 1, 2019, with an estimated study completion date of April, 3, 2023. Protocol version 1.5 was written on April 2, 2019, and approved by the relevant ethical committee (CPP Nord-Ouest I) on April 26, 2019.

## Discussion and conclusion

NECT has shown effectiveness in reducing self-stigma in patients with psychotic-related disorders in a range of contexts in previous research. Considering the prevalence of moderate to high self-stigma in SMI and its impact on functional outcomes, NECT appears to be a promising intervention to complete the basket of services proposed by psychiatric rehabilitation centres (cognitive and social cognitive remediation, psychoeducation, social skills training, group cognitive behaviour therapy, vocational rehabilitation, peer-support [36]). If shown to be effective on social function and for different SMI, it is likely that NECT will have widespread implications for clinical practice.

## Data Availability

The study protocol is available online on Clinical Trials: https://clinicaltrials.gov/ct2/show/NCT03972735?term=nect&rank=1
